# Low prevalence of spectacle use in the Hungarian Roma population indicates unmet health needs

**DOI:** 10.1038/s41598-022-07880-3

**Published:** 2022-03-09

**Authors:** Gergely Losonczy, Peter Piko, B. Jeroen Klevering, Zsigmond Kosa, Janos Sandor, Roza Adany

**Affiliations:** 1 Department of Ophthalmology, Donders Institute for Brain, Cognition and Behaviour, Radboud University Medical Center, Nijmegen, The Netherlands; 2 MTA-DE Public Health Research Group, Department of Public Health and Epidemiology, Faculty of Medicine, University of Debrecen, Debrecen, Hungary; 3 Department of Methodology for Health Visitors and Public Health, Faculty of Health, University of Debrecen, Nyíregyháza, Hungary; 4 Department of Public Health and Epidemiology, Faculty of Medicine, University of Debrecen, Debrecen, Hungary

**Keywords:** Disease prevention, Health care economics, Health policy, Health services, Public health, Vision disorders

## Abstract

The Roma population is the largest transnational ethnic minority group in Europe, often facing socioeconomic inequalities and various health problems. In the present study, we investigated visual acuity and its influencing factors along with spectacle use of the Roma population in comparison with the general population in Hungary. A cross-sectional survey was carried out including 832 participants aged 20–64 years. We recorded the uncorrected visual acuity along with anthropometric, demographic, socioeconomic and health-related data of each individual. Although the average uncorrected visual acuity was somewhat higher, the use of a visual aid was significantly less frequent in the Roma population, especially in the group with a visual acuity below 0.5 in both eyes (14.3% vs. 77.1%, *p* < 0.001). Age, abdominal obesity and disturbances of carbohydrate metabolism had a negative impact on visual acuity in both populations; however, the latter was a much stronger risk factor in the Roma population (OR 5.789, 95% CI 2.239–14.964, *p* < 0.001) than in the general population (OR 2.075, 95% CI 1.097–3.926, *p* = 0.025). Our results show serious unmet health needs within the Roma population, which calls for public health programs to improve poor primary care indicators on regular eye examination and much more rigorous diabetes control.

## Introduction

The European Roma population moved from South Asia into Europe between the ninth and fourteenth centuries. Currently, they are the largest transnational ethnic minority group in Europe, with a population of approximately 10–12 million people^1^. Most of the European Union’s Roma population lives in Eastern European countries, including Romania, Bulgaria, Hungary and Slovakia, where they make up between 5 and 10 percent of the population, but there are also sizeable Roma minorities in Western Balkan countries, as well as in Spain, Italy, France and the UK^2–4^. In comparison with the general population, Roma populations have poorer health and a high burden of both communicable and noncommunicable diseases^5–13^, which can be attributed to social exclusion negatively influencing their access to educational, social and health services^14,15^.

A diminished visual function can deeply affect the quality of life, social participation and economic opportunity. Vision impairment also carries a high financial cost for health care services. Vision and hearing impairment have been associated with a greater risk of injuries, loss of functional activity, anxiety and depression^16–20^*.* It has been shown that minority ethnic groups such as Blacks and South Asians in England^21^ and American Chinese people in the US have a higher prevalence of visual impairment than White people^22,23^ and have less access to appropriate health care services and treatments.

The only data available on the visual function of Roma people compared to the general population come from a study conducted in Spain. The Roma population was more likely to present with vision limitations for both far and near sights and showed a lower use of corrective aids than the general population. These findings were associated with poor mental health and lower social participation^24^. The position of the Roma on the labor market is very unfavourable^25^, and their integration is not only a moral but also an economic imperative in the European Union with workforce shortages^26^. Among measures to overcome labor market barriers faced by Roma exploring and fulfilling their unmet health needs is of crucial importance.

In the framework of a complex (behavior and health) survey as part of physical examination, we investigated the visual acuity and spectacle dependence of the Hungarian Roma in comparison with the general population. The aim of the current study was to define uncorrected visual acuity and the use of glasses and/or contact lenses in the Roma population compared to the Hungarian population. We also investigated the effect of demographic, socioeconomic and health parameters on visual acuity in these groups.

## Materials and methods

In 2018, we performed a cross-sectional survey in two northeastern Hungarian counties (Hajdú-Bihar and Szabolcs-Szatmár-Bereg) where the majority of Roma live, frequently in segregated colonies^27^. This study had three main pillars as questionnaire-based, physical, and laboratory investigations involving adults aged 20–64 years from the Hungarian general (HG) and Hungarian Roma (HR) populations. Altogether, 832 participants were randomly selected, including 417 HG (185 male and 232 female) and 415 HR (108 male and 307 female) subjects. Details of sampling, data collection and management are thoroughly described elsewhere^28^. In brief, segregated colonies exceeding 100 inhabitants were identified previously by Roma field workers, and the ethnicity of the colony population was assessed by self-declaration. After verification of a previously created database, twenty colonies were randomly chosen, and twenty-five households were randomly drawn from each colony. Individuals aged 20 to 64 years were identified in each Roma household, and eventually one person was selected by a random table. Individuals aged 20 to 64 years living in private households and registered by general practitioners in the same counties were randomly chosen from the General Practitioners’ Morbidity Sentinel Stations Program’s registry (which has been working continuously since 1998) and served as a reference sample^29^. In addition to anthropometric (among them waist circumference), demographic (age, sex), socioeconomic (among them educational level), and health-related data (as the prevalence of hypertension, diabetes, anti-hypertensive, anti-diabetic and lipid-lowering treatments), fasting blood samples (native and EDTA-anticoagulated) were also collected for routine laboratory tests including—among others—fasting glucose, high-density-lipoprotein-cholesterol (HDL-C), triglyceride (TG) measurements. As previously described in detail for the definition of metabolic syndrome, the following physical and laboratory parameters were considered abnormal by adopting the International Diabetes Federation’s cut-off values: waist circumference: ≥ 94 cm for males and ≥ 80 cm for females; systolic BP of ≥ 130 mmHg and/or diastolic BP of ≥ 85 mmHg; fasting glucose level: ≥ 5.6 mmol/L; fasting triglyceride level: ≥ 1.7 mmol/L; fasting high-density cholesterol (HDL-C) level: < 1.03 mmol/L in males and < 1.29 mmol/L in females^30^.

As part of the physical examination pillar of the survey, uncorrected visual acuity (UCVA) was determined by the corresponding GP with the use of a standard decimal visual acuity chart (Snellen-type Kettesy chart) on each participating individual. Data measured on Roma subjects (n = 310) were compared with the findings obtained on subjects representing the Hungarian general population (n = 396). Decreased UCVA was defined as below 0.5. The use of spectacles or contact lenses was recorded in the questionnaire-based pillar of the study.

### Statistical analysis

All statistical tests were performed using IBM SPSS (version 26, IBM Company, Armonk, NY, USA) software. Average values were compared by the Mann–Whitney U test, while prevalence data obtained in the survey were compared by χ2 test. Multivariate linear and logistic regression analyses were applied to examine the relationship between visual acuity and relevant demographic (education—in binary logistic model as education level is higher or not than primary one, sex) and health parameters (blood lipid levels, abdominal obesity, fasting glucose level and/or antidiabetic treatment, blood pressure and/or antihypertensive treatment). In general, the conventional *p*-value of 0.05 was applied.

### Ethics declarations

All procedures performed in studies involving human participants were carried out by the ethical standards of the institutional and national research committee and with the 1964 Helsinki declaration and its later amendments. This study was approved by the Ethical Committee of the University of Debrecen, Medical Health Sciences Centre (reference No. 2462-2006) and by the Ethics Committee of the Hungarian Scientific Council on Health (reference No. 61327-2017/EKU). All participants provided written informed consent to participate in the study. This article does not contain any studies with animals performed by any of the authors.

## Results

### Main characteristics of the study populations

Age and sex characteristics along with the educational level of the study populations are summarized in Table 1. Only subjects with full records were considered in the analysis.

**Table 1:** Main characteristics of the Hungarian general (HG) and Roma (HR) populations by sex.

	Male		*p*-value	Female		*p*-value
	HG	HR		HG	HR	
	(n = 178)	(n = 71)		(n = 218)	(n = 239)	
	Prevalence (n)			Prevalence (n)		
**Age groups**						
20–34 years	22.5% (40)	23.9% (17)	0.343	28.0% (61)	31.0% (74)	0.041
35–49 years	44.4% (79)	35.2% (25)		33.5% (73)	41.4% (99)	
50–64 years	33.1% (59)	40.8% (29)		38.5% (84)	27.6% (66)	
**Highest educational level**						
Not completed primary school	3.4% (6)	19.7% (14)	< 0.001	1.8% (4)	23.0% (55)	< 0.001
Completed primary school	16.4% (29)	63.4% (45)		21.6% (47)	62.8% (150)	
Completed secondary school	63.3% (112)	16.9% (12)		57.8% (126)	13.0% (31)	
Higher education	16.9% (30)	0.0% (0)		18.3% (40)	0.4% (1)	
N/A	0.6% (1)	0.0% (0)		0.5% (1)	0.8% (2)	

### Uncorrected visual acuity

The average uncorrected visual acuity (UCVA) of all eyes was 0.92 (95% CI 0.88–0.96) in the Hungarian general population and 1.03 (95% CI 0.99–1.08) in the Hungarian Roma population, indicating a statistically significant difference (*p* = 0.001) between them. However, it is important to note that no significant difference was measured in the proportion of eyes with a decreased uncorrected visual acuity defined as UCVA below 0.5 in the two groups (Table 2).

**Table 2:** Average uncorrected visual acuity (A) and the prevalence of severely decreased visual acuity (defined as UCVA < 0.5) (B) in the Hungarian general and Roma populations by sex.

A	Male		*p*-value	Female		*p*-value
	HG	HR		HG	HR	
	Average (95%CI)			Average (95%CI)		
Left eye	0.93 (0.87–0.98)	1.14 (1.04–1.24)	0.001	0.91 (0.86–0.96)	1.01 (0.96–1.07)	0.004
Right eye	0.94 (0.88–0.99)	1.08 (0.98–1.19)	0.052	0.91 (0.86–0.96)	1.00 (0.95–1.06)	0.011
All eyes	0.93 (0.88–0.99)	1.11 (1.02–1.21)	0.009	0.91 (0.86–0.96)	1.01 (0.95–1.06)	0.012

B	HG	HR	*p*-value			
	Prevalence (n)					
Left eye < 0.5	10.9% (43)	8.7% (27)	0.343			
Right eye < 0.5	10.4% (41)	10.0% (31)	0.878			
One eye < 0.5	3.5% (14)	5.2% (16)	0.364			
Both eyes < 0.5	8.8% (35)	6.8% (21)				

### The use of visual aids

There was a statistically significant difference in the proportion of people regularly wearing glasses or contact lenses to correct their far sight. A total of 23.5% and 8.9% of people wore a vision correction aid in the Hungarian general and Roma populations, respectively (*p* < 0.001). The difference was statistically significant for both sexes (male: 21.9% vs. 5.6%, *p* = 0.002; female: 24.9% vs. 9.9%, *p* < 0.001) in each age group (Table 3A). These data indicate that the proportion of people in all age and sex groups making use of a visual aid is significantly less frequent in the Hungarian Roma sample than in the Hungarian general population. The difference in the use of visual aids was independent of visual acuity. Both in the group of people with one or two eyes with a visual acuity of at least 0.5 and in the group of people with a visual acuity below 0.5 on both eyes, the number of people using glasses or contact lenses was significantly lower in the Hungarian Roma population than in the Hungarian general population. The most striking difference was found in the group with a visual acuity below 0.5 in both eyes (14.3% vs. 77.1%, *p* < 0.001), indicating a serious unmet health need in the Hungarian Roma population (Table 3B).

**Table 3:** Frequency of people using glasses or contact lenses in the Hungarian general (HG) and Roma (HR) populations by age group (A) and representation of individuals using glasses or contact lenses with uncorrected visual acuity of ≥ 0.5 vs. < 0.5 in the Hungarian general (HG) and Roma (HR) populations (B).

	HG	HR	*p*-value
	Prevalence (n)		
**A**			
20–34 years	22.8% (23)	4.4% (4)	< 0.001
35–49 years	17.8% (27)	5.6% (7)	0.002
50–64 years	31.5% (45)	18.9% (18)	0.032
**B**			
Both eyes ≥ 0.5	15.9% (55)	8.4% (23)	0.006
One eye < 0.5	92.9% (13)	18.8% (3)	< 0.001
Both eyes < 0.5	77.1% (27)	14.3% (3)	< 0.001

### The effect of different health parameters on visual acuity

We examined the effect of demographic and cardiometabolic risk factors on uncorrected visual acuity using linear regression analysis and found that Roma ethnicity was correlated with slightly better visual acuity in the whole cohort, while age, abdominal obesity and elevated fasting glucose level and/or antidiabetic treatment had a negative influence on the visual acuity values in the entire cohort as well as in both populations. Female sex was a significant risk factor only among Roma. We could not find any statistically significant correlation between visual acuity values and blood lipid parameters in any of the populations (Table 4).

**Table 4:** The correlation obtained by linear regression analysis between visual acuity (average visual acuity for both eyes, left and right eyes as a continuous outcome variable) and cardiometabolic risk factors/outcomes as components of metabolic syndrome, as well as demographic characteristics on combined (A), Hungarian general (B) and Roma (C) populations.

	Average of left and right eyes			Left eye			Right eye		
	Beta	95% CI	*p*-value	Beta	95% CI	*p*-value	Beta (95% CI)	95% CI	*p*-value
**A—combined population**									
Age	− 0.006	− 0.009 to − 0.004	< 0.001	− 0.006	− 0.008 to − 0.003	< 0.001	− 0.006	− 0.008 to − 0.003	< 0.001
Roma ethnicity*	0.092	0.027 to 0.157	0.006	0.110	0.044 to 0.176	0.001	0.079	0.013 to 0.146	0.019
Female sex*	− 0.039	− 0.100 to 0.022	0.210	− 0.048	− 0.109 to 0.014	0.128	− 0.034	− 0.096 to 0.028	0.283
Education level*	0.005	− 0.028 to 0.037	0.784	0.002	− 0.031 to 0.035	0.900	0.005	− 0.028 to 0.039	0.751
Abdominal obesity*	− 0.100	− 0.168 to − 0.033	0.003	− 0.098	− 0.166 to − 0.030	0.005	− 0.101	− 0.169 to − 0.032	0.004
Reduced HDL-C level*	0.025	− 0.035 to 0.085	0.411	0.027	− 0.034 to 0.088	0.381	0.029	− 0.032 to 0.090	0.355
Elevated TG level and/or lipid-lowering treatment*	0.039	− 0.024 to 0.101	0.225	0.034	− 0.030 to 0.097	0.299	0.042	− 0.022 to 0.106	0.197
Elevated fasting glucose level and/or anti-diabetic treatment*	− 0.142	− 0.208 to − 0.076	< 0.001	− 0.148	− 0.214 to − 0.081	< 0.001	− 0.134	− 0.201 to − 0.067	< 0.001
Elevated blood pressure and/or anti-hypertensive treatment*	0.039	− 0.022 to 0.099	0.207	0.036	− 0.025 to 0.097	0.251	0.031	− 0.031 to 0.092	0.329
**B—Hungarian general population**									
Age	− 0.004	− 0.007 to − 0.001	0.010	− 0.004	− 0.007 to − 0.001	0.016	− 0.003	− 0.006 to − 0.001	0.024
Female sex*	0.001	− 0.072 to 0.073	0.988	− 0.002	− 0.074 to 0.071	0.967	− 0.007	− 0.079 to 0.064	0.843
Education level*	0.026	− 0.017 to 0.068	0.232	0.023	− 0.019 to 0.066	0.281	0.029	− 0.013 to 0.071	0.173
Abdominal obesity*	− 0.110	− 0.195 to − 0.025	0.011	− 0.106	− 0.192 to − 0.021	0.015	− 0.109	− 0.193 to − 0.025	0.011
Reduced HDL-C level*	0.039	− 0.039 to 0.117	0.322	0.040	− 0.038 to 0.119	0.313	0.037	− 0.040 to 0.114	0.348
Elevated TG level and/or lipid-lowering treatment*	0.014	− 0.065 to 0.092	0.729	0.003	− 0.076 to 0.082	0.939	0.020	− 0.058 to 0.097	0.619
Elevated fasting glucose level and/or anti-diabetic treatment*	− 0.098	− 0.180 to − 0.015	0.021	− 0.100	− 0.184 to − 0.017	0.019	− 0.088	− 0.170 to − 0.006	0.036
Elevated blood pressure and/or anti-hypertensive treatment *	0.049	− 0.026 to 0.123	0.202	0.045	− 0.030 to 0.120	0.243	0.048	− 0.026 to 0.122	0.207
**C—Roma population**									
Age	− 0.011	− 0.015 to − 0.006	< 0.001	− 0.010	− 0.014 to − 0.006	< 0.001	− 0.010	− 0.015 to − 0.006	< 0.001
Female sex*	− 0.131	− 0.239 to − 0.023	0.018	− 0.151	− 0.261 to − 0.041	0.007	− 0.101	− 0.214 to 0.012	0.080
Education level*	− 0.031	− 0.082 to 0.020	0.228	− 0.033	− 0.085 to 0.018	0.206	− 0.033	− 0.087 to 0.020	0.219
Abdominal obesity*	− 0.066	− 0.175 to 0.043	0.234	− 0.066	− 0.177 to 0.045	0.241	− 0.070	− 0.185 to 0.044	0.225
Reduced HDL-C level*	0.003	− 0.091 to 0.096	0.957	0.007	− 0.088 to 0.103	0.879	0.011	− 0.087 to 0.109	0.832
Elevated TG level and/or lipid-lowering treatment*	0.070	− 0.033 to 0.173	0.185	0.071	− 0.034 to 0.175	0.185	0.074	− 0.033 to 0.182	0.176
Elevated fasting glucose level and/or anti-diabetic treatment*	− 0.220	− 0.326 to − 0.113	< 0.001	− 0.230	− 0.338 to − 0.122	< 0.001	− 0.211	− 0.323 to − 0.099	< 0.001
Elevated blood pressure and/or anti-hypertensive treatment*	0.056	− 0.047 to 0.158	0.185	0.052	− 0.052 to 0.15	0.324	0.037	− 0.071 to 0.144	0.502

*Binary covariate.

Logistic regression analysis was also used to analyze the effect of demographic and cardiometabolic risk factors on the proportion of people with an uncorrected visual acuity below 0.5 in both eyes. Disturbance of carbohydrate metabolism was the only factor showing any association with UCVA in this analysis (Table 5). Notably, elevated fasting glucose levels and/or antidiabetic treatment were a much stronger risk factor in the Roma population (OR 5.789, 95% CI 2.239–14.964, *p* < 0.001) than in the Hungarian general population (OR 2.075, 95% CI 1.097–3.926, *p* = 0.025).

**Table 5:** The correlation obtained by logistic regression analysis between severe visual impairment (visual acuity for both eyes less than 0.5) and cardiometabolic risk factors/outcomes as components of metabolic syndrome, as well as demographic characteristics in combined (A), Hungarian general (B) and Roma (C) populations**.**

	Less than 0.5 for both eyes		
	OR	95% CI	*p*-value
**A—combined population**			
Age	1.031	1.007–1.055	0.011
Roma ethnicity*	0.860	0.484–1.528	0.606
Female sex*	1.372	0.800–2.355	0.250
Education level*	1.210	0.935–1.565	0.147
Abdominal obesity*	1.334	0.692–2.571	0.389
Reduced HDL-C level*	0.605	0.347–1.055	0.077
Elevated TG level and/or lipid-lowering treatment*	0.842	0.488–1.452	0.537
Elevated fasting glucose level and/or anti-diabetic treatment*	2.760	1.646–4.626	< 0.001
Elevated blood pressure and/or anti-hypertensive treatment*	1.166	0.664–2.048	0.593
**B—Hungarian general population**			
Age	1.022	0.995–1.050	0.111
Female sex*	1.007	0.542–1.870	0.983
Education level*	1.047	0.725–1.511	0.806
Abdominal obesity*	1.253	0.576–2.726	0.570
Reduced HDL-C level*	0.502	0.242–1.040	0.064
Elevated TG level and/or lipid-lowering treatment*	1.170	0.609–2.251	0.637
Elevated fasting glucose level and/or anti-diabetic treatment*	2.075	1.097–3.926	0.025
Elevated blood pressure and/or anti-hypertensive treatment*	1.075	0.556–2.076	0.831
**C—Roma population**			
Age	1.064	1.015–1.116	0.010
Female sex*	5.369	1.283–22.462	0.021
Education level*	1.434	0.999–2.059	0.051
Abdominal obesity*	1.130	0.312–4.096	0.853
Reduced HDL-C level*	0.768	0.297–1.987	0.586
Elevated TG level and/or lipid-lowering treatment*	0.438	0.154–1.246	0.122
Elevated fasting glucose level and/or anti-diabetic treatment*	5.789	2.239–14.964	< 0.001
Elevated blood pressure and/or anti-hypertensive treatment*	1.332	0.441–4.021	0.611

*Binary covariate.

We further investigated the effect of interaction between covariates and ethnicity on the visual acuity using logistic regression analysis. Of all the covariates used in statistical calculations Roma ethnicity showed a significant interaction with elevated fasting blood glucose and/or anti-diabetic treatment. This interaction presented a statistically significant risk for a visual acuity below 0.5 in both eyes. (OR 1.905, 95% CI 1.395–2.726, *p* < 0.001) (Table 6).

**Table 6:** The association of covariates with Roma ethnicity on visual acuity as a ≥ 0.5 vs. < 0.5 in both eyes categorical outcome variable in the interaction model.

	Interaction with Roma ethnicity		
	OR	95% CI	*p*-value
Age	1.006	0.997–1.016	0.204
Female sex*	1.101	0.875–1.384	0.412
Education level*	1.093	0.938–1.275	0.255
Abdominal obesity*	1.129	0.771–1.652	0.533
Reduced HDL-C level*	0.750	0.531–1.058	0.101
Elevated TG level and/or lipid-lowering treatment*	0.828	0.576–1.189	0.307
Elevated fasting glucose level and/or anti-diabetic treatment*	1.950	1.395–2.726	< 0.001
Elevated blood pressure and/or anti-hypertensive treatment*	1.082	0.767–1.526	0.654

*Binary covariate.

## Discussion

We conducted a three pillars (questionnaire-based, physical, and laboratory investigations) cross-sectional study in the Hungarian general and Roma populations in 2018 in Northeast Hungary on samples of randomly selected adults aged 20–64 years. We defined uncorrected visual acuity and the use of glasses and/or contact lenses as well as the effect of demographic, socioeconomic and health parameters on visual acuity. The only data available on the visual function of Roma came from Spain and showed that this population was more likely to present with vision limitations and showed a lower use of corrective aids than the general population. Contrary to this study, we found that the Hungarian Roma population had a slightly but significantly better average uncorrected visual acuity in every age and sex group than the Hungarian general population. However, this difference did not translate into a clinically significant difference, as the proportion of people with a visual acuity below 0.5 did not significantly differ in the two populations. It is noteworthy that the educational level was significantly lower in the Roma population in all age groups, which could have a great role in their socioeconomic segregation and lower social opportunities. However, an association between more years spent in education and myopia, as suggested by a previous study^31^, could not be confirmed in the present study, as educational level had no statistically significant effect on visual acuity. We cannot exclude the possibility that refractive parameters in the Roma population are closer to normal emmetropy, which could be a genetically determined characteristic^32,33^. Cultural and social isolation, as well as a high level of genetic consanguinity throughout the centuries, has led to a certain genetic segregation of Roma populations^34–36^. This is characterized not only by an overload of founder mutations but also by the lack or decreased frequency of some other pathogenic mutations in these populations. These factors lead to decreased genetic diversity in the Roma population, but the frequency of rare genetic variants and pathogenic alleles maybe both higher^37–39^ and lower^16,40^ compared with the general population.

One of the strongest differences between the two populations was found in the use of vision correcting aids. The use of simple visual aids for distance vision, such as glasses or contact lenses, was significantly lower in members of the Hungarian Roma population. This difference was confirmed in all ages and both sex groups. This difference could not be explained through the somewhat better visual acuity measured in the Roma population because the proportion of eyes with a visual acuity below 0.5 did not significantly differ between the two groups. It is noteworthy that the largest difference in wearing a vision correction aid was found in the group with a visual acuity below 0.5 with one or both eyes, indicating a serious unmet health need in the Hungarian Roma population. The lack of proper visual correction in the Roma population indicates that they do not receive adequate refractive and/or ophthalmic care. This finding is in harmony with the results from other studies showing limited access of Roma to health services (both preventive and curative) in general^2,9,13,41–44^. This may severely contribute to their lower education level, lower social opportunities and lower access to the labor market and finally to their social segregation.

We examined the association of demographics and certain health parameters with visual acuity. Age, abdominal obesity and elevated fasting glucose level and/or antidiabetic treatment had a statistically significant negative effect on visual acuity as a continuous variable. However, when visual acuity was analyzed as a binary covariate and decreased visual acuity was defined as vision on both eyes less than 0.5, the only statistically significant association was found with the elevated fasting glucose level and/or anti-diabetic treatment in both populations. Elevated fasting glucose and/or antidiabetic treatment was a stronger risk factor among the Roma population than in the Hungarian general population. In line with this finding, we have also shown that the interaction between Roma ethnicity and disorders of carbohydrate metabolism significantly increase the risk of a visual acuity below 0.5 in both eyes. This is in line with our previous findings indicating that elevated plasma glucose or known type 2 diabetes are significantly more frequent in the Hungarian Roma population than in the Hungarian general population and that the proportion of untreated diabetes is very high (53.3%) in the Roma population^45,46^. Additionally, the proportion of missed glucose check-ups was significantly higher in the Roma population than in the de Hungarian population^47^. It is worth mentioning that in a comparative analysis, the Hungarian general population carried a greater genetic risk for the development of type 2 diabetes mellitus than Roma, but in a combined population model, the effect of ethnicity was relatively strong on the development of diabetes (OR 2.484, *p* < 0.001)^48^.

The prevalence of metabolic syndrome (HG: 39.8%, HR: 44.0%) and insulin resistance (HG: 42.3% and HR: 40.5%) was almost equally very unfavorable in both populations^28^. In our present study, there was a trend for people with higher waist circumference to have lower visual acuity, as seen in the linear regression analysis using visual acuity as a continuous variable. However, this association could not be verified when using a clinically significant cut–off value (UCVA lower than 0.5), and vision was considered a binary variable. On the other hand, based on the present study, one cannot exclude the influence of central obesity on visual acuity in these populations. This should be further investigated, not only because very little is known about this association but also because the prevalence of abdominal obesity is strongly increasing in the Hungarian Roma population^46^.

In the Roma population, female sex was shown to be an independent risk factor for lower visual acuity; however, this should be taken into consideration with precaution, as the female sex was overrepresented in the Roma sample, which can be seen as a limitation of the study. This cross-sectional survey was based on randomly selected households, and as we described previously in many households, a proportionally higher number of women were at home during the day when most visits took place, and men were at work at another location^28^. The Hungarian government quadrupled the budget for public works between 2010 and 2015 for all Hungarian municipalities. This is especially relevant for villages in the northeastern region of Hungary, where segregated Roma settlements are concentrated, and our study was carried out. The majority of workers participating in the program are men from deprived Roma communities.

Our results suggest a slightly better average uncorrected visual acuity in the Hungarian Roma population but a much lower use of vision aids as well as a stronger negative effect of diabetes on uncorrected visual acuity in comparison with the Hungarian general population. These results call for public health actions for the improvement of poor primary care indicators on regular eye examination^49^ and much more rigorous diabetes control within the Roma population^47^ in Hungary. These measures could have an important impact on the social integration of Roma people in Hungarian society.

## Data Availability

The datasets used in the current study are not publicly available due to privacy/ethical restrictions but are available from the corresponding senior author on reasonable request.
